# Rationale for the Use of Topical Calcineurin Inhibitors in the Management of Oral Lichen Planus and Mucosal Inflammatory Diseases

**DOI:** 10.7759/cureus.74570

**Published:** 2024-11-27

**Authors:** Michael B Chancellor

**Affiliations:** 1 Biotechnology, Lipella Pharmaceuticals, Pittsburgh, USA; 2 Urology, Oakland University William Beaumont School of Medicine, Auburn Hills, USA

**Keywords:** inflammation, liposomes, mouth, oral cancer, oral lichen planus, oral medicine, saliva, tacrolimus

## Abstract

Oral lichen planus (OLP) is a chronic inflammatory condition that affects the mucous membranes of the oral cavity and is characterized by a T-cell-mediated autoimmune response. It presents a therapeutic challenge due to its relapsing nature, causing significantly decreased quality of life and, in some cases, increasing the risk of malignant transformation. While topical corticosteroids have long been the first-line therapy for OLP, their long-term use is associated with adverse effects, such as mucosal atrophy and candidiasis. This has driven interest in alternative therapies, particularly topical calcineurin inhibitors (TCIs), such as tacrolimus, which offer a steroid-sparing approach.

This review explores the pathophysiological basis of OLP, examines the role of TCIs in its treatment, and evaluates emerging therapies, with a specific focus on the use of a topical liposomal formulation of tacrolimus. These formulations aim to achieve high local drug concentrations while minimizing systemic absorption. OLP is a complex and multifactorial disease that requires a multifaceted approach to management. While current therapies provide symptomatic relief, there is a need for more effective and safer treatment options.

Emerging therapies, including advanced drug delivery systems, biologics, and alternative therapies, hold promise for improving the management of OLP. Future research should focus on identifying novel therapeutic targets and developing strategies that can achieve sustained remission with minimal side effects.

## Introduction and background

Introduction

Oral lichen planus (OLP) is a chronic autoimmune condition that affects the mucous membranes of the oral cavity. Certain medications, including nonsteroidal anti-inflammatory drugs (NSAIDs), antihypertensives, and gold injections, can trigger OLP [1].

OLP is characterized by white or red patches, erosions, and ulcerations on the tongue, cheeks, gums, and other areas of the mouth. While OLP is not considered a malignant condition itself, it has a significant risk of malignant transformation, particularly in its erosive or ulcerative forms [1-5].

Common symptoms of OLP include pain, burning, and discomfort in the affected areas. Lesions may cause pain while brushing teeth or wearing dentures, leading to poor oral hygiene. In severe cases, OLP can result in debilitating pain that hampers speaking, eating, and swallowing, potentially causing malnutrition, weight loss, and breathing difficulties. In addition to physical discomfort, OLP can lead to anxiety, depression, social isolation, and a decreased quality of life, impacting patients' ability to work and engage socially [1,5].

Epidemiology

Lichen planus is a common dermatological condition, with approximately 60% of patients presenting with oral manifestations. OLP affects approximately 1-2% of the global population, particularly middle-aged adults, with an estimated prevalence of over six million cases in the U.S. [4,6]. The chronic nature of OLP and its potential for malignant transformation necessitate long-term management, for which topical calcineurin inhibitors (TCIs) offer a valuable alternative to traditional therapies. OLP primarily affects women aged 30 to 60 years [7-9]. The widespread use of corticosteroids in this population highlights the need for safer long-term options, such as TCIs, which reduce the risk of steroid-induced side effects. Risk factors for OLP include genetic predisposition, environmental triggers, and chronic stress, which can exacerbate the immune response. The immune modulation provided by TCIs offers a targeted strategy to address these factors.

The prevalence of OLP ranges from 1% to 2%, with a female-to-male ratio of 2:1 and an age of onset between 30 and 60 years [3]. Diagnosis is based on clinical appearance and biopsy findings [1]. OLP can be categorized into three clinical subtypes: reticular, atrophic or erythematous, and erosive and/or ulcerative. Unlike cutaneous lichen planus, OLP typically has a chronic course with little chance of spontaneous resolution [5].

OLP and oral cancer

The significant burden of OLP and its association with malignant transformation underscores the need for effective treatment and careful monitoring of affected individuals. Currently, available therapies are primarily palliative rather than curative [5]. The rate of malignant transformation in OLP may exceed that of other oral mucosal diseases. Risk factors for malignant transformation include disease duration, extent of involvement, and the presence of ulceration, erythema, or hyperkeratosis [8].

The American Academy of Oral Medicine (AAOM) acknowledges that patients with OLP have an increased risk of developing oral cancer and require careful management and monitoring by trained clinicians [9]. Although definitive long-term prospective studies are lacking, accumulated data from case series and systematic reviews suggest a cancer development rate of approximately 0.5% to 1% among OLP patients, which is significantly higher than the rate reported in the general population [10-14]. A recent systematic review reported an overall malignancy rate of 1.09% [7]. The most common sites of oral cancer in OLP patients include the tongue, buccal mucosa, and gingiva.

Pathophysiology and etiology

The pathogenesis of OLP involves an immune-mediated response characterized by the activation of cytotoxic CD8+ T-cells, which target basal keratinocytes, leading to apoptosis and the formation of characteristic mucosal lesions. This immune response is driven by the overexpression of pro-inflammatory cytokines, such as TNF-α and IFN-γ [1,15-17].

The calcineurin pathway is critical for T-cell activation and cytokine production. TCIs inhibit calcineurin, preventing the nuclear factor of activated T-cells (NF-AT) from translocating to the nucleus, thereby blocking the transcription of pro-inflammatory cytokines. This mechanism directly addresses the immune dysregulation central to OLP.

Genetic susceptibility, particularly specific HLA alleles, has been associated with OLP. However, the immune response remains the primary driver of the disease process. Environmental triggers, including stress, dental materials, and certain medications, may exacerbate OLP by stimulating or perpetuating the immune response in genetically predisposed individuals, making immune modulation through TCIs a rational therapeutic approach.

Inflammation and saliva cytokines in OLP

The inflammatory changes associated with OLP involve a complex interaction of cytokines, including IL-1, IL-6, TNF-α, and IFN-γ, which sustain the inflammatory response (Table 1) [16,17]. By inhibiting cytokine production through calcineurin blockade, TCIs directly target the inflammatory milieu that perpetuates OLP. Rhodus et al. [17] investigated the potential for detecting cytokine levels in whole unstimulated saliva to monitor the therapeutic effects of topical dexamethasone in erosive OLP patients. The study included 13 OLP patients and an equal number of age- and sex-matched controls. Following dexamethasone treatment, the levels of TNF-α, IL-1α, IL-6, and IL-8 significantly decreased, with the IL-1α and IL-8 levels returning to those of the controls. These preliminary results suggest that salivary analysis of NF-kappaB-dependent cytokines may be valuable for monitoring therapeutic responses in patients with OLP.

**Table 1:** Key cytokines implicated in oral lichen planus (OLP) OLP: oral lichen planus

Cytokine	Role in OLP
IL-1	Involved in T-cell activation and keratinocyte apoptosis
IL-6	Regulates immune responses and is associated with disease severity
TNF-α	Mediates inflammation and immune responses
IFN-γ	Promotes T-cell activation and perpetuates the immune response

Clinical presentation

OLP manifests with a range of symptoms, including white, lacy patches, erosions, and ulcerative lesions, often accompanied by a burning sensation. Ulcerative and erosive forms of OLP are the main reasons for symptoms and cause significant pain and discomfort, for which immunosuppressive therapy is recommended. Topical TCIs may be particularly useful in ulcerative/erosive and atrophic subtypes, which tend to be refractory to steroids and are prone to chronic relapses [6,18-20].

The diagnosis of OLP is primarily clinical and is based on the characteristic appearance of the lesions. However, a biopsy may be needed to confirm the diagnosis and rule out other conditions such as leukoplakia, candidiasis, and squamous cell carcinoma. Histopathological examination typically reveals band-like infiltration of lymphocytes in the subepithelial layer and degeneration of the basal cell layer [1,3,8,10,16].

## Review

Current therapeutic approaches

Topical corticosteroids remain the most commonly used medications for OLP, acting to reduce inflammation and suppress the immune response in affected areas. Various corticosteroids, including triamcinolone acetonide, fluocinonide, and clobetasol propionate, can be administered as mouth rinses, gels, or ointments [6,16,21]. TCIs, such as tacrolimus and pimecrolimus, serve as alternatives and work to suppress the immune response and reduce inflammation [18,22].

A 2015 systematic review and meta-analysis indicated that clobetasol and tacrolimus improved OLP symptoms, with odds ratios of 1.19 and 8.00, respectively [23]. A 2020 (Table 2) Cochrane review further suggested that topical corticosteroids might be more effective in alleviating OLP pain than placebo, whereas tacrolimus appeared superior to corticosteroids for pain resolution [6].

**Table 2:** Current treatment options for oral lichen planus (OLP) OLP: oral lichen planus

Treatment category	Specific treatment	Mechanism of action	Advantages	Limitations
Topical corticosteroids	Dexamethasone elixir or solution; prednisolone solution; budesonide solution; clobetasol solution or gel; betamethasone gel; fluocinolone gel	Anti-inflammatory by inhibiting cytokines	Efficacy in reducing inflammation	Risk of mucosal atrophy with prolonged use
Systemic corticosteroids	Prednisone	Suppresses systemic immune response	Control of severe or widespread OLP	Systemic side effects, not suitable for long-term use
Topical immunomodulators	Tacrolimus, pimecrolimus	Suppress T-cell activity	Effective in steroid-resistant cases	Potential for local irritation
Systemic immunosuppressants	Cyclosporine, azathioprine	Inhibits immune cell proliferation	Used in refractory cases	Risk of systemic immunosuppression, side effects
Adjunctive therapies	Antimicrobials (e.g., tetracycline), analgesics	Reduce secondary infection, relieve pain	Helps manage symptoms, reduces discomfort	Does not address underlying inflammation
Natural therapies	Aloe vera, curcumin	Potential anti-inflammatory, antioxidant effects	Fewer side effects, potential complementary use	Limited clinical evidence, variable effectiveness
Phototherapy	Low-level laser therapy, photodynamic therapy	Modulates immune response, promotes healing	Non-invasive, can be used adjunctively	Requires specialized equipment, variable efficacy

In a 2022 meta-analysis, no significant differences in clinical resolution, pain alleviation, or relapse rates were found between tacrolimus and corticosteroids. Although tacrolimus was associated with a higher incidence of minor, transient adverse effects, these did not hinder continued use [19]. Another 2022 network meta-analysis noted that, compared with placebo, tacrolimus outperformed corticosteroids in clinical response and symptom reduction [24]. In 2023, a network meta-analysis found that TCIs had comparable efficacy to topical corticosteroids, with only calcineurin inhibitors showing statistically significant clinical resolution compared to placebo, albeit with a higher rate of adverse effects [15].

Utz et al. [20] demonstrated that 97% of patients treated with a medical oral-rinse formulation of tacrolimus achieved objective remission after 24 months, with minimal side effects. The most common adverse reaction to topical tacrolimus is a transient burning or stinging sensation; other side effects may include dysgeusia, mucosal paresthesia, pruritus, headache, and nausea [15,19,20,22-25].

Currently, most available formulations of topical tacrolimus are in ointment form, which may be poorly tolerated for oral lesions. The efficacy of topical treatments is often limited by challenges in maintaining adequate contact time on moist mucosal surfaces (Table 3).

**Table 3:** Considerations with topical therapy and drug delivery in the oral cavity

Issue	Description	Impact on treatment
Barriers to drug penetration	The keratinized mucosa can limit the penetration of drugs into deeper tissues	Reduced drug efficacy in reaching target tissues
Irritation and sensitivity	Some topical agents can cause local irritation, burning, or hypersensitivity reactions	Discomfort leading to reduced patient adherence
Difficulty in adherence	Patients may struggle with frequent application of topical therapies	Poor compliance, incomplete treatment courses
Unpleasant taste	Some formulations may alter taste or cause unpleasant sensations	Decreased patient willingness to use the therapy
Oral drug retention	Topical agents may be washed away by saliva or disrupted by eating/drinking	Shorter drug exposure, necessitating reapplication
Formulations	Oral cavity conditions require formulations that can adhere to mucosal surfaces	Increased complexity in drug formulation and cost
Saliva dilution	Saliva can dilute topical medications, reducing drug concentration and efficacy	Reduced drug contact time, lower treatment effectiveness
Risk of systemic absorption	Potential for systemic absorption of drugs, especially with potent steroids	Possible systemic side effects, complicating treatment

The absence of a cure, established standards of care, and Food and Drug Administration (FDA)-approved therapies for OLP underscores the need for new, safe, and effective treatment options. Evidence supports the use of tacrolimus, particularly for patients who are resistant to or intolerant of other therapies.

Topical therapies

Topical corticosteroids are the mainstay of treatment for OLP, providing significant relief from inflammation and symptoms. Agents such as clobetasol and betamethasone are commonly used. However, long-term use can lead to mucosal atrophy and candidiasis (Table 4). Vathanasanti et al. [26] compared the effectiveness of fluocinolone acetonide 0.01% and dexamethasone 0.1% mouthwash in treating symptomatic OLP patients. Thirty-four patients (27 females and 7 males; mean age: 47.26±11.78 years) with symptomatic OLP were treated for six weeks with either fluocinolone acetonide 0.01% mouthwash or dexamethasone 0.1% mouthwash in a randomized, double-blind, clinical trial. Pain severity and lesion size and severity were assessed via the visual analog scale (VAS) pain score and clinical score, respectively. At the end of the treatment period, pain symptoms (VAS pain score) and lesion size and severity (clinical score) were significantly lower in the fluocinolone acetonide 0.01% and dexamethasone 0.1% mouthwash groups than in the baseline group. However, the difference in these scores between the groups was not significant. The authors concluded that both fluocinolone acetonide 0.01% and dexamethasone 0.1% mouthwash were effective in treating symptomatic OLP. Villa et al. [27] highlighted the importance of an effective vehicle for drug delivery in treating OLP, demonstrating that a 0.1 mg/mL dexamethasone solution in Mucolox was more effective for managing OLP compared to dexamethasone solution alone at the same concentration.

**Table 4:** Risks with topical steroid use for oral lichen planus (OLP)

Risk/problem	Description	Clinical consequence
Fungal infection	Fungal infection due to immunosuppression in the oral cavity	Oral thrush, requiring antifungal treatment
Masking underlying infections	Steroids can mask signs of bacterial or viral infections	Delayed diagnosis and treatment of coexisting infections
Mucosal atrophy	Thinning of the oral mucosa due to prolonged steroid use	Increased susceptibility to trauma, discomfort
Burning and irritation	Local discomfort upon application of potent topical steroids	Decreased patient compliance
Systemic absorption	Risk of steroids being absorbed into the bloodstream	Potential systemic side effects (e.g., adrenal suppression)
Steroid resistance	Reduced efficacy over time or in certain patients	Necessitates alternative or adjunctive therapies
Rebound inflammation	Worsening of symptoms upon discontinuation of steroids	Challenges in tapering off treatment, prolonged therapy

Topical immunomodulators, including tacrolimus and pimecrolimus, are used as alternative treatments, particularly in patients who are resistant to steroids. These agents work by suppressing T-cell activation, thereby reducing inflammation. TCIs are effective in cases where the immune response plays a dominant role, as indicated by histopathological findings.

Systemic therapies

Systemic corticosteroids, such as prednisone, are reserved for severe or widespread cases of OLP. While effective, their use is limited by the risk of systemic side effects, including immunosuppression and adrenal suppression. In refractory cases, immunosuppressants such as cyclosporine and azathioprine may be used. These agents inhibit immune cell proliferation, reducing the autoimmune response. However, they carry significant risks, including increased susceptibility to infections.

Adjunctive therapies: antimicrobials and analgesics

Antimicrobial agents, such as tetracycline, may be used to manage secondary infections, whereas analgesics can help alleviate pain. Lifestyle modifications, including stress management and smoking cessation, are also recommended.

Limitations of current therapies

Current therapies for OLP have shown effectiveness in controlling symptoms, but they are not without limitations. The use of drugs is empiric without controlled regulatory trials. There are no specific topical or systemic drugs approved by the FDA for the indication of OLP. The long-term use of topical corticosteroids, although most commonly used, can lead to adverse effects, such as mucosal atrophy and increased risk of infection (Table 5). Moreover, some patients may develop resistance to corticosteroids, necessitating alternative treatments.

**Table 5:** Gaps in current treatment of oral lichen planus (OLP) OLP: oral lichen planus

Gap	Description	Impact on patient care
Side effects of long-term steroid use	Prolonged use of topical/systemic steroids leads to adverse effects	Mucosal atrophy, systemic complications
Steroid resistance	Some patients do not respond adequately to corticosteroids	Limits treatment options, necessitates alternatives
Lack of targeted therapies	Broad-spectrum immunosuppressant versus T-cell specific	Risk of systemic side effects, non-specific action
Long-term efficacy	Many treatments are effective short term, but relapse is common after discontinuation	Difficulty in achieving sustained remission
Patient compliance	Long-term treatments can be cumbersome, leading to poor adherence	Incomplete treatment courses, reduced efficacy
Insufficient data on alternative therapies	Natural and complementary therapies lack robust clinical evidence	Uncertainty in effectiveness, hesitance in use
Limited options for refractory cases	Patients with severe or resistant OLP have few effective treatment options	Leads to persistent symptoms, decreased quality of life

Emerging and promising therapies

Recent advancements in drug delivery systems have led to the development of novel topical formulations for OLP [28]. These include nanoformulations, bioadhesive gels or patches [29], and liposomal delivery systems that enhance drug retention and absorption in the oral mucosa. Biologic agents that target specific immune pathways, such as TNF-α inhibitors (e.g., etanercept) and B-cell-depleting therapies (e.g., rituximab), are being explored for the treatment of OLP. These agents offer the potential for more targeted and effective management of the disease. Low-level laser therapy (LLLT) and photodynamic therapy (PDT) have shown promise in managing OLP. These noninvasive treatments modulate the immune response and promote healing of the mucosal lesions. Natural products, such as curcumin and aloe vera, are being investigated as alternative or adjunctive therapies for OLP. These agents possess anti-inflammatory and antioxidant properties, suggesting a potential complementary approach to conventional treatments.

Future directions

Overall, there is a great unmet need for safe and effective treatments for OLP and other oral mucosal diseases. The gaps in current treatments are summarized in Table 5 [28]. Research into the pathogenesis of OLP is likely to yield new therapeutic targets and strategies. Clinical trials are currently exploring the efficacy of novel biologics, advanced drug delivery systems, and natural products in the management of OLP.

Topical tacrolimus for OLP and oral mucosal inflammation

Tacrolimus is a potent immunosuppressant that has been approved by the FDA for systemic use in preventing transplant rejection and as a topical ointment for moderate to severe atopic dermatitis. It acts by inhibiting IL-2-dependent T-cell activation and has a direct inhibitory effect on cell-mediated immunity [30]. Topical tacrolimus may be an optimal option for managing chronic inflammatory and autoimmune conditions in the oral cavity because of its effectiveness and fewer long-term side effects than steroids. While topical steroids are highly effective for short-term management, their long-term use is less favorable because of significant side effects. Other immunomodulators offer potential benefits but lack the extensive data and established efficacy seen with tacrolimus and steroids, limiting their use owing to uncertainty about long-term outcomes.

Tacrolimus (also known as FK-506; trade name Prograf®, Advagraf®, Protopic®) is a macrolide antibiotic that acts primarily on T-helper cells, although some inhibition of suppressor and cytotoxic T-cells also occurs [26]. By binding to the intracellular protein FKBP-12, tacrolimus inhibits calcineurin, a calcium and calmodulin-dependent phosphatase. This process inhibits the translocation of the NF-AT family of transcription factors, leading to reduced transcriptional activation of early cytokine genes, including interleukin (IL)-2, interferon-γ, tumor necrosis factor (TNF)-α, IL-3, IL-4, CD40L, and granulocyte-macrophage colony-stimulating factor. Ultimately, this results in reduced proliferation of lymphocytes. Although the mode of action of tacrolimus is similar to that of cyclosporine, tacrolimus has been shown to be less nephrotoxic [31-33].

While systemic administration carries a high risk of adverse events, such as nephrotoxicity and hypertension due to arterial constriction, topical administration to dermal inflammatory sites has a low incidence of adverse events [34]. Because tacrolimus is a highly lipophilic drug, formulating it for topical administration has been challenging and is a key rationale for the development of a liposomal tacrolimus formulation (LP-10), which is under development for the treatment of OLP [35].

Topical liposomal tacrolimus clinical experience

Liposomal formulations of tacrolimus have been explored to enhance drug delivery and retention at mucosal surfaces. In a study evaluating a liposomal tacrolimus preparation (LP-10), patients with refractory moderate to severe sterile hemorrhagic cystitis showed that intravesical administration of LP-10 was well tolerated and demonstrated signs of clinical efficacy [36]. These findings support the potential use of liposomal tacrolimus formulations for localized inflammatory conditions.

Topical liposomal tacrolimus for OLP

A multicenter, dose-ranging trial evaluating the safety, tolerability, and efficacy of LP-10 in patients with symptomatic OLP is currently being conducted in the United States [35]. This study includes adult male and female subjects (≥18 years old) with symptomatic OLP. Approximately 24 subjects will be enrolled at multiple sites. The study will evaluate the safety, tolerability, and efficacy of LP-10 at doses of 0.25 mg, 0.5 mg, and 1.0 mg of tacrolimus. The treatment phase includes administration of a 10 mL LP-10 oral rinse for three minutes twice a day for four weeks. The follow-up phase includes one post-treatment visit two weeks after the last oral LP-10 dose. The results from the study are anticipated in 2025.

Discussion

The management of OLP remains challenging owing to the chronic nature of the disease and the limitations of current therapies. While topical corticosteroids are effective for short-term management, their long-term use is associated with significant risks. Emerging therapies, including new topical formulations, biologics, and phototherapy, offer hope for more effective and safer treatment options. As research continues to unravel the molecular pathways involved in OLP, new opportunities for targeted therapies are likely to emerge.

The introduction of TCIs represents a paradigm shift in the management of OLP. Their ability to modulate the immune response without the adverse effects of corticosteroids makes them a rational choice for long-term management. While emerging therapies such as biologics and laser treatments are promising, TCIs remain a practical and effective option for most patients.

## Conclusions

OLP is a complex and multifactorial disease that requires a multifaceted approach to management. While current therapies provide symptomatic relief, there is a need for more effective and safer treatment options. Emerging therapies, including advanced drug delivery systems, biologics, and alternative therapies, hold promise for improving the management of OLP. Future research should focus on identifying novel therapeutic targets and developing strategies that can achieve sustained remission with minimal side effects.

TCIs offer a targeted, steroid-sparing alternative for the treatment of OLP, addressing the underlying immune dysfunction without the risks associated with long-term steroid use. Continued research into their optimal use, including novel formulations and delivery systems, will further enhance their role in the management of OLP.
